# Transcranial Electric Stimulation Can Impair Gains during Working Memory Training and Affects the Resting State Connectivity

**DOI:** 10.3389/fnhum.2017.00364

**Published:** 2017-07-12

**Authors:** Annie Möller, Federico Nemmi, Kim Karlsson, Torkel Klingberg

**Affiliations:** Department of Neuroscience, Karolinska Institutet Stockholm, Sweden

**Keywords:** transcranial direct current stimulation (tDCS), working memory training, fMRI, resting state functional connectivity, transcranial random noise stimulation (tRNS)

## Abstract

Transcranial electric stimulation (tES) is a promising technique that has been shown to improve working memory (WM) performance and enhance the effect of cognitive training. However, experimental set up and electrode placement are not always determined based on neurofunctional knowledge about WM, leading to inconsistent results. Additional research on the effects of tES grounded on neurofunctional evidence is therefore necessary. Sixty young, healthy, volunteers, assigned to six different groups, participated in 5 days of stimulation or sham treatment. Twenty-five of these subjects also participated in MRI acquisition. We performed three experiments: In the first one, we evaluated tES using either direct current stimulation (tDCS) with bilateral stimulation of the frontal or parietal lobe; in the second one, we used the same tDCS protocol with a different electrode placement (i.e., supraorbital cathode); in the third one, we used alternating currents (tACS) of 35 Hz, applied bilaterally to either the frontal or parietal lobes. The behavioral outcome measure was the WM capacity (i.e., number of remembered spatial position) during the 5 days of training. In a subsample of subjects we evaluated the neural effects of tDCS by measuring resting state connectivity with functional MRI, before and after the 5 days of tDCS and visuo-spatial WM training. We found a significant impairment of WM training-related gains associated with parietal tACS and frontal tDCS. Five days of tDCS stimulation was also associated with significant change in resting state connectivity revealed by multivariate pattern analysis. None of the stimulation paradigms resulted in improved WM performance or enhanced WM training gains. These results show that tES can have negative effects on cognitive plasticity and affect resting-state functional connectivity.

## Introduction

Transcranial electric stimulation (tES) is a relatively newly rediscovered tool that both the scientific and general society hope can enhance the performance of several cognitive skills (Dubljević et al., 2014). The most commonly used stimulation type is anodal transcranial direct current stimulation (tDCS), which is assumed to depolarize the resting potential and thereby increasing excitability in neurons underlying the anode, (Nitsche et al., 2008). The supposed mechanism of action is modifying intrinsic brain activity by an increased probability of action potentials (Polanía et al., 2011; Weber et al., 2014).

Using tES over repeated sessions while training cognitive skills could affect intrinsic brain activity and interact with training-induced plasticity. Indeed, enhanced training gains have been documented for numerical competence (Cohen Kadosh et al., 2010) and vocabulary learning (Meinzer et al., 2014). Likewise, a positive effect of tDCS on working memory (WM) training gain was found by Au et al. (2016) after 7 days of training, although results from other groups reported the effect of tDCS during WM training as being limited to on-line effect (Martin et al., 2013) or near-transfer measures (Richmond et al., 2014). An obvious difference between the abovementioned studies is that while Au et al. (2016) trained visuo-spatial WM (VSWM), Martin et al. (2013) and Richmond et al. (2014) trained verbal WM. However, we argue that the previous studies combining tDCS with WM training (Martin et al., 2013; Richmond et al., 2014; Au et al., 2016) have limited grounding in neurofunctional knowledge about WM. They all placed a single anode over the left or right frontal lobe. However, most neuroimaging studies show bilateral activation during performance of VSWM tasks (Rottschy et al., 2012). Hence, it is reasonable to think that bilateral stimulation could be more effective than unilateral, when combining tDCS and VSWM training. Moreover, studies of WM training have consistently shown changes in activity in the parietal lobe (Olesen et al., 2004; Constantinidis and Klingberg, 2016). Therefore, we also included groups that received parietal stimulation. In the light of the above considerations, we decided to use bilateral stimulation of either the frontal or parietal lobe, which has not been investigated in previous WM studies.

The supposed mechanism of tDCS through increase in probability of action potentials could, through long term potentiation (LTP), lead to an increased functional connectivity in the networks affected by stimulation (Polanía et al., 2011; Weber et al., 2014). Since earlier studies of WM training alone have suggested that strengthened functional connectivity is a mechanism that underlies the gain in WM capacity (Jolles et al., 2013; Kundu et al., 2013; Astle et al., 2015), we hypothesized that tDCS over either the parietal or frontal lobes during WM training would increase the strengthening of connectivity within the fronto-parietal network compared to WM training alone. We therefore acquired resting state fMRI data as a part of our first experiment, to evaluate the effect of a combination of tDCS and WM training on changes in connectivity.

Evaluating the results of our first experiment, we found that both the group receiving frontal tDCS, and the one receiving parietal, had lower WM training gain than the control group, receiving sham stimulation. This effect was in the opposite direction as our hypothesis, and led us to question our setup. Specifically, we focused on the position of the cathode. Looking at previous studies, a variety of cathode positions have been used, e.g., extracephalic, supraorbital, and contralateral. Some authors suggest that the cathode is passive (Clemens et al., 2013), whilst others argue that it has an important effect in itself, either through altering the distribution and strength of the currency field generated by the anode (Bikson et al., 2010), or through decreased excitability in neurons underlying the cathode (Nitsche and Paulus, 2000). To evaluate the possibility that the placement of the cathodes affected the results of the stimulation in the first experiment, we conducted a second experiment, using supraorbital cathodes instead of occipital, and only stimulating the DLPFC bilaterally.

As moving the cathode did not lead to enhancement of the training effect, we tested the hypothesis that a different type of stimulation could enhance the effect of WM training. Specifically, we turned to transcranial alternating current stimulation (tACS). Unlike tDCS, the effects of tACS are thought to be caused by altering the intrinsic oscillations of the brain (Herrmann et al., 2013). This is achieved by alternating the direction of the stimulation current with a predefined frequency, thereby synchronizing stimulated brain areas (Zaehle et al., 2010). This technique has been used to influence motor and sensory processes, but also higher cognitive functions (see Herrmann et al., 2013, for review). For example, both Polanía et al. (2012) and Jaušovec and Jaušovec (2014) have shown that tACS in a theta frequency can enhance performance of WM. However, the choice of a stimulation frequency is not obvious since different aspects of WM have been linked to ranges in both the theta, alpha and gamma bands (Howard et al., 2003; Roberts et al., 2013; Roux and Uhlhaas, 2014; Honkanen et al., 2015). The WM task in our study is highly focusing on the WM load, i.e., the number of items required to be held in memory during the delay. E.g., no interfering components are introduced to increase difficulty and the responses given are deemed either right or wrong, no judge of quality of the memory is made. The load component of WM has specifically been linked to gamma frequency in humans (Howard et al., 2003; Basar-Eroglu et al., 2007). Moreover, different means to artificially entrain brain oscillations are used experimentally, e.g., in animals. Kim et al. (2016) used optogenetic stimulation and showed that stimulation in the frontal cortex at 30–40 Hz frequency enhances attention in mice. Based on this we chose to stimulate the frontal or parietal cortex bilaterally with tACS at a frequency of 35 Hz in the third experiment of this study. During the progress of the present study, other groups have also published works indicating that tACS in similar frequencies can have an effect on cognitive performance (Hoy et al., 2015; Santarnecchi et al., 2016).

## Results and Methods

### Experiment 1

#### Methods

##### Subjects

Thirty healthy adults were divided into three groups before the baseline session, and were blinded to which type of stimulation they received; “Sham stimulation” (*n* = 10, six males, mean age = 29.3, *SD* = 2.9), “Frontal tDCS – Occipital Cathodes” (*n* = 10, five males, mean age = 29.3, *SD* = 6.3) and “Parietal tDCS” (*n* = 10, seven males, mean age = 28.2, *SD* = 2.9). All participants had to meet the following inclusion criteria: no neurological or psychiatric disorders, no psychoactive medications, no metal objects implanted, no abuse of drugs or alcohol, and no previous experience of tES. The study was approved by the local ethical committee in Stockholm and all participants gave informed written consent according to the declarations of Helsinki.

##### Procedure

**FIGURE 1:**
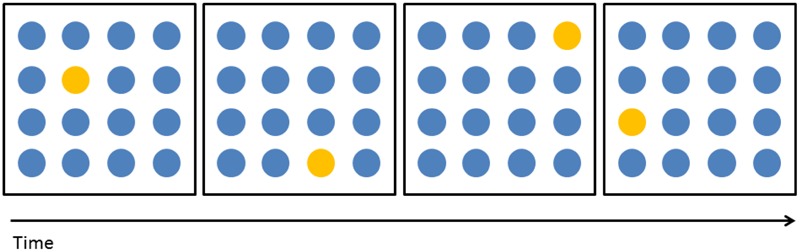
Training task. During each trial participants were presented with a sequence of N dots (N = level of difficulty) that changed color in a specific order. Sixteen dots were presented in a 4-by-4 grid. Each dots changed color for 1000 ms and then the following dot would immediately change color. After the end of the probe the task of participants was to repeat the presented sequence, in the same order, by touching the correct dots. **Figure 1** shows an example of a sequence at level 4.

During five sessions, participants performed WM training on consecutive days for ∼25 min while receiving tDCS. The training consisted of an adaptive VSWM task: remembering and repeating a presented sequence of dots in a 4-by-4 grid, one of the tasks previously used in Nemmi et al. (2016). Difficulty was adapted by changing the number of to-be-remembered dots in the sequence (hereafter “level”) (**Figure 1**). The daily outcome measure was the mean of the levels of the three highest-level items correctly performed during the training session. Note that subjects participating in the imaging arm of the study were submitted to the MRI acquisition protocol during one session the week before the beginning of the training (baseline session) and during the fifth day of training. These subjects received tDCS stimulation without simultaneous WM training during the baseline session, just before entering the MRI scanner. This procedure was chosen so that any difference between the MRI measures at day 5 and at baseline can safely be ascribed to the combination of tDCS and cognitive training during the 5-day treatment rather than to the short-term effect of a single tDCS session.

##### Stimulation

**FIGURE 2:**
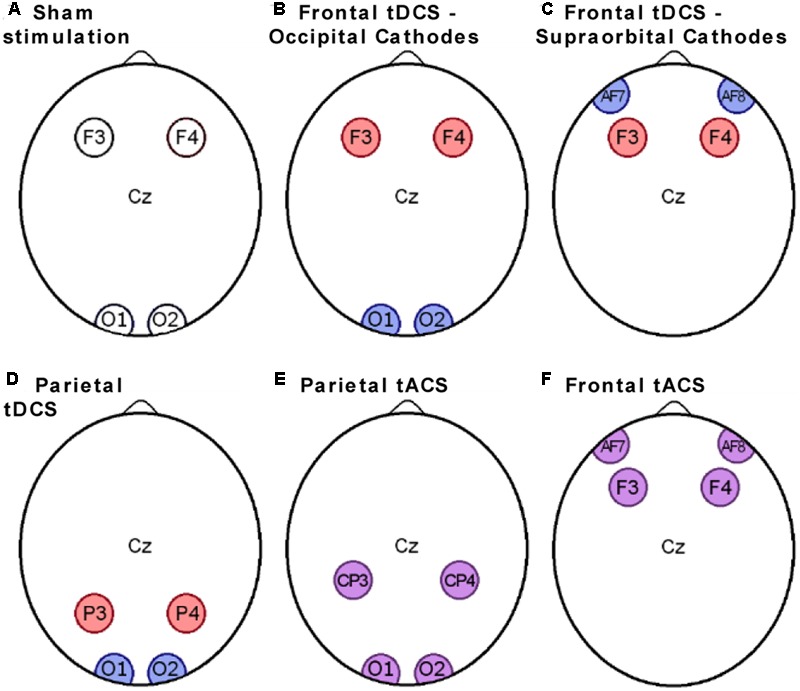
Electrode placements. The scalp electrodes in the six experimental groups were placed in positions according to the 10–20 international system. Passive electrodes are shown in white, anodes in red, cathodes in blue, and electrodes with alternating current in purple. **(A)** Electrodes placement for the sham group; **(B)** Electrodes placement for the Frontal tDCS - Occipital cathodes group; **(C)** Electrodes placement for the Frontal tDCS - Supraorbital Cathodes group; **(D)** Electrodes placement for the Parietal tDCS group; **(E)** Electrodes placement for the Parietal tACS group; **(F)** Electrodes placement for the frontal tACS group.

Direct current was generated by a StarStim stimulator, using 25 cm^2^ circular electrodes. Impedance was monitored and stimulation programs were controlled using the Neuroelectrics Instrument Controller software (both from Neuroelectrics Barcelona SL). For the “Sham stimulation” and “Frontal tDCS – Occipital Cathodes” groups the anodes were placed in F3 and F4 positions (**Figures 2A,B**), while for the “Parietal tDCS” group they were in the P3 and P4 positions according to the 10–20 international system (**Figure 2D**). Cathodes were always in O1 and O2 in Experiment 1 and two grounding electrodes were placed below the right ear. Active stimulation groups received 1 mA current (ramped up during 30 s and down during 30 s), while sham stimulation consisted of stimulation ramped up and down within 30 s, as previously been described (Gandiga et al., 2006).

##### Statistical analysis

Working memory performance was analyzed using a mixed linear model with Day (1–5, treated as a continuous variable), Group (Frontal, Parietal, or Sham) and Day × Group interaction as fixed effects. Both intercept and slope of the subjects were treated as random effects, in order to account for repeated measures. Statistical analyses of training data was performed in R (R Core Team, 2015). The mixed linear model was implemented using the package nlme (Pinheiro and Bates, 2000); the analysis of variance table was extracted using the anova.lme function in the same package, using type 3 sum of squares. *F* test and *p*-values for the Wald test are reported. Whenever the interaction between the factors Day and Group was significant, we performed planned comparisons between experimental groups (active stimulation) and sham.

Finally, the effect of Day within each group was investigated fitting a mixed effect models with performance as dependent variable and Day as independent variable, intercept and slope of each subject was treated as random effect. For these models, we report standardized betas and *p*-values.

##### MRI acquisition

The first 25 subjects participated in an additional MR scanning session (“Sham stimulation” *n* = 8; “Frontal tDCS – Occipital Cathodes” *n* = 9; “Parietal tDCS” *n* = 8). During this first session, as well as the last, participants first went through tDCS (either active or sham) during rest and then an MRI scan. MRI data was acquired on a 3T MRI scanner (Discovery General Electric), using a structural T1 weighted sequence (resolution = 0.94 mm × 0.94 mm, TE = 2.5 ms, TR = 5.7 ms, TI = 400 ms, FoV = 24 cm, 180 axial slices, flip angle of 12°) and a 10 min resting state fMRI sequence (eyes open, fixating white cross; 42 axial slices, 3.0 mm slice thickness, 0.5 mm slice gap, TR 2000 ms). Data were analyzed using CONN functional connectivity toolbox (v14.p) (Whitfield-Gabrieli and Nieto-Castanon, 2012) in SPM8 (Welcome Trust Center of Neuroimaging, University College London, United Kingdom). Preprocessing included non-brain tissue removal, slice-timing correction, realignment, segmentation of structural images and normalization to the Montreal Neurological Institute (MNI) template. Functional volumes were spatially smoothed using a Gaussian 8 mm kernel and individually band-pass-filtered at 0.008–0.09 Hz in the temporal domain. Noise correction was performed using CompCor (Behzadi et al., 2007), that regresses out from the functional time-series the first two principal components of the time-series extracted from white matter and CSF. Moreover, six movement nuisance regressors and their time derivatives plus their quadratic values were regressed out from the BOLD time-series. Images that were regarded as movement outliers (defined as overall movement of >2 mm or root mean squared change in bold signal from volume to volume > 9) were regressed out, using the ART toolbox^1^. After preprocessing we performed a Voxel-to-Voxel connectivity analysis. In this analysis, the pairwise connectivity pattern between each voxel and the rest of the brain (all other voxels) is computed. After that, a dimensionality reduction step is implemented by means of principal component analysis (PCA maximizing the explained inter-subject variability in the resulting patterns using a lower number of spatial components; here we calculated five components but only retained the first, which accounted for 34% of the variance). We then performed an analysis looking at associations between this component (i.e., the weight of the first principal component) and interaction between group and time (i.e., we looked for voxels were the component was different among groups and between sessions) (Whitfield-Gabrieli et al., 2016). The *p*-value for this analysis was set to 0.05, corrected with FDR.

#### Results

##### Behavioral data

**FIGURE 3:**
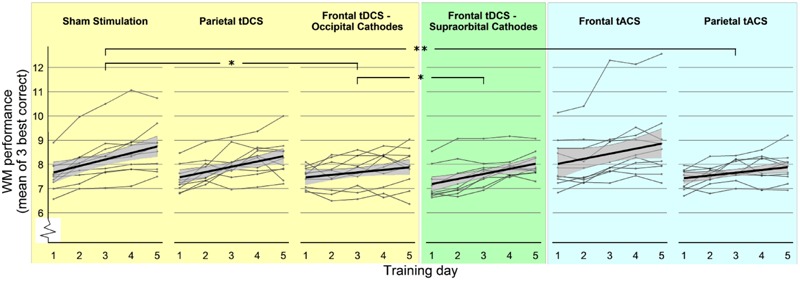
WM performance over the five training days. Participants’ performance in WM training throughout the training period, experimental groups separated. Each thin line represents one participant, showing daily mean of the three highest-level correct items as dots. Thick lines represent the regression line of the group, surrounded by the 95% confidence interval (gray area). Stars (^∗^*p* < 0.05; ^∗∗^*p* < 0.01) refer to significant effects of interaction between Group and Day in mixed linear models comparing two groups (see Results).

The main effect of Group was not significant [*F*(2,27) = 0.22, *p* = 0.80], indicating that there was no overall effect of stimulation on WM performance. The effect of Day was significant [*F*(1,117) = 71.24, *p* < 0.001], indicating that there was a training effect in all groups. Crucially, there was a significant interaction between Group and Day on WM performance, *F*(2,117) = 4.39, *p* = 0.014, indicating that the groups differed significantly in their improvement (**Figure 3**). As a *post hoc* analysis, we performed planned pairwise mixed models. The interaction between Group and Day on WM performance was significant for the comparison of “Sham stimulation” vs. “Frontal tDCS – Occipital Cathodes” [*F*(1,78) = 6.71, *p* = 0.011] with significantly lower gain in the frontal group, but it did not reach significance for “Sham stimulation” vs. “Parietal tDCS,” *F*(1,78) = 0.83, *p* = 0.36. These results thus showed that frontal tDCS did not affect average performance, but significantly impaired training gains. The effect of Day was comparable in the Sham group (β = 0.4, *p* < 0.001) and the Parietal tDCS group (β = 0.45, *p* < 0.001) and lower in the in the Frontal tDCS – Occipital Cathode group (β = 0.22, *p* = 0.021).

##### Resting state connectivity

**FIGURE 4:**
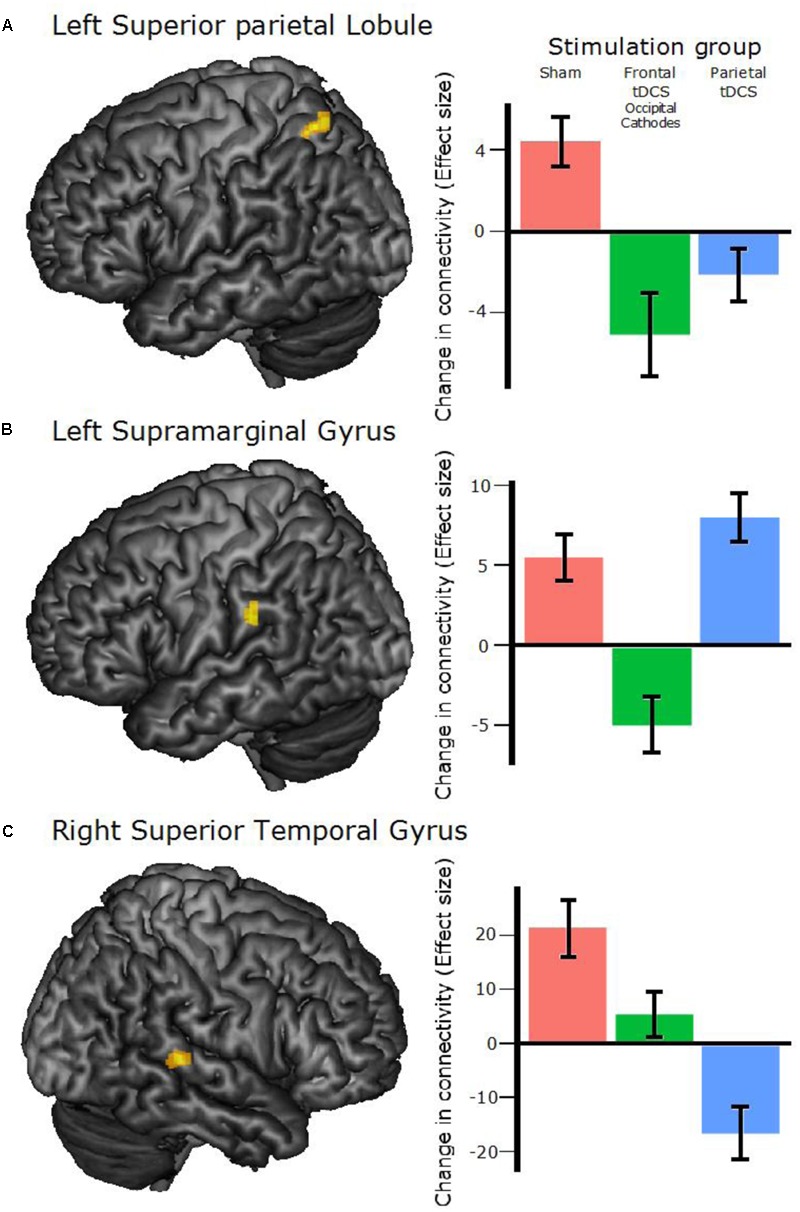
Clusters found to differ between groups and between pre–post scans. **(A)** The left Superior Parietal Lobule cluster, peak coordinates: –24 –60 +60 (all coordinates are in MNI space). **(B)** The left Supramarginal Gyrus cluster, peak coordinates: –50 –30 +26. **(C)** The right Superior Temporal Gyrus cluster, peak coordinates: +52 –34 +06. **Right panel**: The bar plots show effect sizes from the voxel-to-voxel multivariate pattern analysis (MVPA) with any Group differences and Post–Pre as contrasts. The bars correspond to the mean of all the voxels within the corresponding cluster and all participants in the specified stimulation group. Error bars show 95% confidence interval. The unit on the *y*-axis is arbitrary.

Calculating the change in connectivity patterns (first principal component weight of each voxel) between Pre and Post scanning sessions and comparing any differences between the three experimental groups: three clusters of voxels showed a significant difference between groups (*p*-value for each cluster < 0.05 was corrected for false discovery rate, *p-*FDR; all coordinates for cluster peaks are in MNI space). Each cluster was superimposed on the average T1 image to identify its anatomical localization. One was located in the left Superior Parietal Lobule (SPL; -24 -60 +60; *n* of voxels = 44; cluster *p*-FDR = 0.02; **Figure 4A**), one in the left Supramarginal Gyrus (SMG; -50 -30 +26; *n* of voxels = 50; cluster *p*-FDR = 0.02; **Figure 4B**) and one in the right Superior Temporal Gyrus (STG; +52 -34 +06; *n* of voxels = 70; cluster *p*-FDR = 0.009; **Figure 4C**).

To evaluate which of the groups differed in these clusters we performed two-tailed *t*-tests as a *post hoc* analysis (**Figure 4**, right panel). These revealed that for the right STG cluster, the connectivity patterns of all three groups differed significantly from each other [Sham vs. Frontal: *t*(15) = 2.39, *p* = 0.03; Sham vs. Parietal: *t*(14) = 5.29, *p* < 0.001; Frontal vs. Parietal: *t*(15) = 3.43, *p* = 0.004], while only two of the group comparisons were significant in the left SMG cluster [Sham vs. Frontal: *t*(15) = 3.69, *p* = 0.002; Sham vs. Parietal: *t*(14) = 1.18, *p* = 0.26; Frontal vs. Parietal: *t*(15) = 3.86, *p* = 0.002] and the left SPL cluster [Sham vs. Frontal: *t*(15) = 5.55, *p* < 0.001; Sham vs. Parietal: *t*(14) = 4.56, *p* < 0.001; Frontal vs. Parietal: *t*(15) = 1.22, *p* = 0.24]. The beta values of this measure are an index of the cluster’s whole-brain connectivity pattern, hence no inference can be made about any increases or decreases in the connectivity. This analysis thus showed that the type of stimulation significantly affected the pattern of resting-state connectivity.

**Table 1** reports the average movement for the three groups and the two times points.

**Table 1:** Means and standard deviations of the framewise displacement (FD) for the three groups included in the imaging arm and for the two time points.

	Sham	tDCS – Frontal	tDCS – Parietal
Pre	0.18 (±0.15)	0.11 (±0.06)	0.09 (±0.02)
Post	0.1 (±0.03)	0.11 (±0.07)	0.08 (±0.02)

*A mixed linear model with FD as dependent variable, and group, time and their interaction as independent variable showed that the groups did not differ in terms of movement, and that the effects of time and the interaction were not significant.*

##### Discussion

The results of this first experiment were in the opposite direction as the one hypothesized: parietal stimulation did not enhance the effect of WM training and frontal stimulation impaired it. While the null effect of the parietal stimulation was not so surprising, as no evidence of the effect of parietal stimulation on WM training has been reported in literature, the results of the frontal group were more troubling, as positive effects have been reported (Martin et al., 2013; Richmond et al., 2014). We turned our attention to the placement of the cathode in our setup, as certain groups have reported a cathodal effect on performance that can be due to alteration in the distribution and strength of the currency field (Bikson et al., 2010), or decreased excitability of the underlying neuron reducing the resting potential (Nitsche and Paulus, 2000). Hence, we conducted a second experiment, investigating if the placement of the cathodes affected the results of the stimulation.

### Experiment 2

Following the result of the first study, 10 additional healthy subjects were recruited. We hypothesized that the detrimental effect of frontal tDCS observed in the first experiment could be an effect of the cathodes’ placement: e.g., cathodes placed onto the occipital cortex could have interfered with the visual processing of the stimulus, thus impairing WM. As such, we decided to move the cathodes onto the supraorbital area.

#### Subjects

Ten subjects were included in a group to investigate a different cathode position for tDCS over the frontal lobes; “Frontal tDCS – Supraorbital Cathodes” (*n* = 10, five males, mean age = 28.6 years, *SD* = 7.2). Inclusion criteria were identical to those of Experiment 1 and the ethical permission was obtained by the same board.

#### Procedure

The procedure was identical to that of Experiment 1. No participant in the second experiment participated in the MRI arm of the study.

#### Stimulation

The stimulation protocol for Experiment 2 was identical to that of Experiment 1, except that the cathodes were placed onto AF7 and AF8 (**Figure 2C**).

#### Statistical Analysis

The analytical strategy was the same as in Experiment 1, but here we added the group “Frontal tDCS – Supraorbital Cathodes” in the model. We fitted a mixed effect model with the same variable as in Experiment 1 and we extracted the ANOVA table.

#### Results

There was no significant main effect of group [*F*(2,27) = 1.31, *p* = 0.29] but there was a main effect of Day [*F*(1,117) = 69.01, *p* < 0.001]. Importantly, the interaction between Group and Day was also significant [*F*(2,117) = 4.29, *p* = 0.016]. This result was followed by *post hoc* pairwise comparisons in mixed models only including two groups at a time. There was no significant interaction between Group and Day on WM performance between the “Sham stimulation” and the new group “Frontal tDCS – Supraorbital Cathodes” [*F*(1,78) = 1.15, *p* = 0.28]. However, there was a significant interaction in the comparison of “Frontal tDCS – Supraorbital Cathodes” and “Frontal tDCS – Occipital Cathodes” [*F*(1,78) = 4.17, *p* = 0.045]. The effect of Day was significant in the “Frontal tDCS – Supraorbital Cathodes” (β = 0.4, *p* < 0.001), comparable to the effect in the Sham group and the Parietal tDCS group.

#### Discussion

These results indicated that the significant negative impact of frontal tDCS in Experiment 1 was due to placement of the cathodes over the occipital lobe. However, the group with supraorbital placement of the cathodes did not differ from the control group receiving sham stimulation, hence this stimulation still did not show the training enhancing effect we predicted.

### Experiment 3

Following the results of the second experiment, we decided to narrow down our hypothesis based on a recent finding in the animal literature. Synchronization of parvalbumin interneurons in the medial PFC of rats to gamma oscillation through optogenetic stimulation improves performance in an attentional task (Kim et al., 2016). Since the supposed effect of tACS is a synchronization of the regions underlying the electrodes to the frequency being delivered (Zaehle et al., 2010; Thut et al., 2011) and in the light of the association between attention and WM (Gazzaley and Nobre, 2012), we wanted to observe if tACS in the gamma frequency applied to the DLPFC or the parietal cortex would enhance WM training.

#### Subjects

We recruited 20 subjects and we assigned them to two groups: “Frontal tACS” (*n* = 10, six males, mean age = 27.9 years, *SD* = 5.8) and “Parietal tACS” (*n* = 10, five males, mean age = 28.4 years, *SD* = 3.8).

#### Procedure

The WM training procedure was identical to the previous experiments. No participant in the third experiment participated in the MRI arm of the study.

#### Stimulation

For the “Frontal tACS” group posterior electrodes were in F3 and F4 positions and anterior electrodes in supraorbital positions, just below AF7 and AF8 (**Figure 2F**). For the “Parietal tACS” group, the anterior electrodes were in CP3 and CP4 positions and the posterior electrodes were in O1 and O2 (**Figure 2E**). The current strength was 1 mA and alternated with a low gamma frequency of 35 Hz. To avoid the appearance of phosphenes, i.e., perception of flashes of light, subjects in the groups receiving tACS were tested starting at 700 μA before beginning the first session, similar to phosphene checks used in other studies (Strüber et al., 2013). No subject reported uncomfortable sensations or light flashes and the current could be ramped up to 1 mA. This test was integrated in the regular impedance check to make the difference in procedure minimal between the groups receiving tDCS/Sham stimulation or tACS.

#### Statistical Analysis

The analytical strategy was the same as in the previous experiments: we fitted a mixed effect model with groups (“Frontal tACS,” “Parietal tACS,” and “Sham”) and the same variable as in Experiment 1 and we extracted the ANOVA table.

#### Results

**FIGURE 5:**
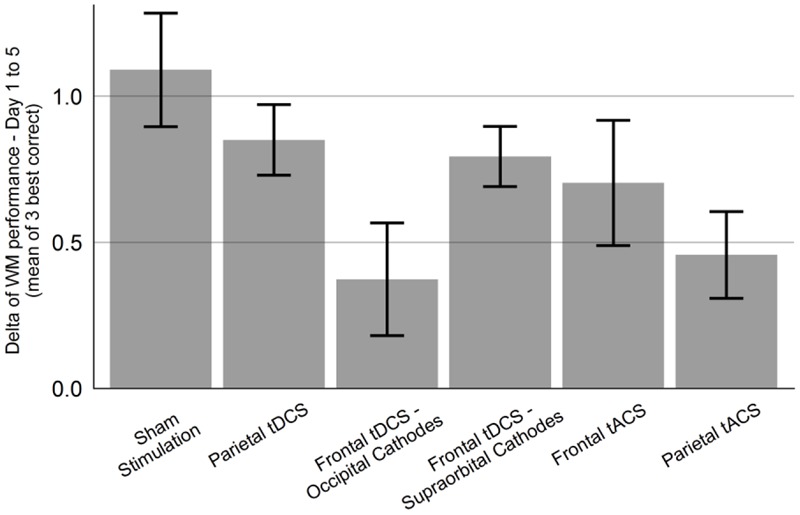
The groups’ difference in WM performance between first and last session. All six experimental groups improved their WM performance from first to last session, measured as mean of the three highest-level correct items of each session. Error bars show standard error of the mean.

The main effect of Group was not significant [*F*(2,27) = 1.61, *p* = 0.22], while the effect of Day was significant [*F*(1,117) = 54.58, *p* < 0.001]. The interaction between Group and Day on WM performance was marginally significant, *F*(2,117) = 2.85, *p* = 0.062. Since the interaction effect showed a trend toward significance, we also performed pairwise mixed models. For the comparison of the “Sham stimulation” vs. “Parietal tACS” groups, there was a significant interaction effect between Group and Day [*F*(1,78) = 7.74, *p* = 0.007], with significantly lower gain in the “Parietal tACS” group (**Figure 3**). The “Sham stimulation” and “Frontal tACS” groups did not differ significantly [*F*(1,78) = 0.63, *p* = 0.43]. Neither of the comparisons revealed a main effect of Group. Parietal tACS thus significantly impaired training gains compared to sham stimulation. The effect of Day was comparable in both the Frontal tACS and the Parietal tACS groups (respectively, β = 0.23, *p* < 0.001 and β = 0.23, *p* < 0.001). **Figure 5** shows the delta values between WM performance on the first and last days to visualize mean training gain in the six different groups (note that delta values were not used in the analysis).

The daily measures, as well as the average of the 5 days, were converted to *z*-scores and analyzed regarding outliers within their respective experimental group. No daily or average score exceeded two standard deviations from the mean. Although no subject’s performance was found to lie outside two standard deviations from the mean, a high performer receiving sham stimulation as well as one in the “Frontal tACS” group might be regarded as outliers on visual inspection (**Figure 3**). Removing these subjects did not change our results significantly in either Experiments 1, 2, or 3.

Means and standard deviations for the demographic variables as well as one-way ANOVA comparing the groups are reported in **Table 2**.

**Table 2:** Means and standard deviations of demographic and cognitive variables.

	Sham stimulation	Frontal tDCS – Occipital Cathodes	Frontal tDCS – Supraorbital Cathodes	Parietal tDCS	Parietal tACS	Frontal tACS	Mean	One-way ANOVA
Age; mean (SD)	29.3 (2.9)	29.3 (6.3)	28.6 (7.2)	28.2 (2.9)	28.4 (3.8)	27.9 (5.8)	28.9 (4.3)	*F*_(5,54)_ = 0.13 *p* = 0.98
Sex; male, female	6, 4	5, 5	5, 5	7, 3	5, 5	6, 4	5.7, 4.3	χ^2^_(5)_ = 1.36 *p* = 0.93
Education; mean (SD)	18.1 (2.2)	16.6 (2.7)	16.5 (3.1)	17.3 (1.8)	16.3 (2.8)	17.4 (3.3)	17.0 (2.6)	*F*_(5,53)_ = 0.62 *p* = 0.68
IQ; mean (SD)	13.5 (2.4)	13.8 (2.2)	13.9 (2.0)	13.1 (1.8)	13.3 (1.5)	15.0 (2.4)	13.8 (2.1)	*F*_(5,53)_ = 1.03 *p* = 0.41
WM performance at day 1; mean (SD)	7.6 (0.6)	7.5 (0.4)	7.2 (0.6)	7.4 (0.5)	7.4 (0.4)	8.1 (1.0)	7.5 (0.7)	*F*_(5,54)_ = 2.25 *p* = 0.06

*There were no significant differences between the six groups regarding age (in years), sex, education (in years) or IQ (as indicated by baseline score on 18 items from Raven’s Standard Progressive Matrices + (Raven, 1998). There was a trend of group difference in WM performance at day 1 (mean of the levels of the three highest-level items correctly performed during the first training session), with the “Frontal tACS” having the highest mean. However, this was taken into account by including performance at the first session in the general mixed model used in all analyses of group × time effects.*

## Discussion

In this study, we aimed to test a combination of tDCS and WM training based on the well-known association between VSWM and the fronto-parietal network (Olesen et al., 2004; Rottschy et al., 2012; Constantinidis and Klingberg, 2016). Thus, we first tested the hypothesis that tDCS applied bilaterally over the frontal or parietal lobes would improve the effect of WM training (Experiment 1). Contrary to our hypothesis, we found that frontal tDCS with occipital cathodes had a significant negative effect on training gain. The tDCS stimulation was associated with changes in resting-state connectivity over the 5 days of stimulation, partly within the fronto-parietal network. Experiment 2, where we moved the cathode from the occipital lobe to the supraorbital area, suggested that the impairment of WM training gain observed in the frontal tDCS in Experiment 1 could be due to the placement of cathodes over the occipital lobe. In Experiment 3, we evaluated tACS over the frontal or parietal lobe. The parietal tACS led to significantly lower training gain. None of the stimulations in Experiments 1, 2, or 3 led to enhanced performance of WM tasks or enhanced gain during WM training.

Regarding enhancement of WM capacity (i.e., independent of the effect on plasticity seen during training), all three experiments failed to find an effect of the factor “Group.” This is in agreement with a number of studies failing to see increases in accuracy or capacity from tDCS during WM tasks in single sessions (Tremblay et al., 2014; Horvath et al., 2015; Hill et al., 2016). However, regarding frontal gamma tACS, both positive (Hoy et al., 2015) and mixed (Santarnecchi et al., 2016) results have been reported from single session studies, in contrast to our results. Moreover, one of the WM training studies using tDCS found an increase in average performance but not in training gain (Martin et al., 2013).

However, the aim of our study was mainly to examine effects on plasticity (i.e., training gain). Two previous studies of WM training found increased gain from tDCS (Richmond et al., 2014; Au et al., 2016), which is in disagreement with our results. Possible reasons why our results differ are differences in experimental setup, such as current strength electrode positions and WM tasks, which is discussed in more details below. The effects of tACS on WM are generally less explored than the effects of tDCS. As mentioned in the introduction, gamma tACS has been hypothesized to enhance plasticity, possibly via its effect on LTP (Fell and Axmacher, 2011). Although LTP is one mechanism that can underlie increased interregional connectivity, it is not necessarily the only mechanism behind WM training gain. From a theoretical viewpoint, the stimulation might be more efficient if it was only applied in the delay phase of the WM task (Tallon-Baudry et al., 1998), which is hard to achieve with present technology in humans. It might be that maintaining a forced gamma oscillation throughout the whole WM process actually interferes with the endogenous interplay between different synchronization frequencies. Studies of theta oscillations in relation to cognitive performance have suggested that the timing of oscillations relative to peaks and troughs of endogenous oscillations are of great importance, and theta phase coupling can reduce reaction times during a delayed letter discrimination task (Polanía et al., 2012). This could be one aspect that is hard to achieve when inducing oscillation artificially, and stimulation might even disrupt endogenous oscillation synchrony (Chander et al., 2016). Theta tACS has also been shown to increase spatial WM capacity when applied to the parietal cortex (Jaušovec and Jaušovec, 2014). In this latter study, theta phase was tailored for each subject by adjusting it for the individual alpha peak frequency, probably increasing the effectivity of stimulation. A positive effect of fronto-parietal theta frequency tACS stimulation has been recently confirmed by Violante et al. (2017), although only on a verbal WM task. As for stimulation in the gamma range, Hoy et al. (2015) found that gamma tACS delivered on the DLPFC is effective in improving the accuracy in a 3-back but not a 2-back WM task. The difference in stimulation site (only frontal in Hoy et al., 2015, fronto-parietal in the present study) and task used (3-back WM task in Hoy et al., 2015, VSWM task in the present study) can explain the lack of positive results in the present study.

In Experiment 1, we found that tDCS affected the change in connectivity from baseline to post-intervention. This reflects not only an effect of stimulation on brain activity, but a specific effect of repeated tDCS, since both MRI acquisitions were made straight after stimulation. This could suggest that tDCS can have long-lasting effects on resting state connectivity. The three clusters of significant group differences were located in the left SPL, left SMG and right STG (**Figure 3**). The SPL region is part of the fronto-parietal network previously found to be associated with WM and attention (Corbetta and Shulman, 2002; Rottschy et al., 2012). Since the outcome used for this analysis is a measure of global connectivity for each voxel, i.e., not retaining spatial specificity of the areas connected to the clusters, one can only speculate on the physiological mechanisms involved. Moreover, the signs of the values of change in connectivity (**Figure 4**, left panel) are not meaningful (i.e., they are the mean weight of the voxels in the cluster on a principal component), so that one cannot interpret the changes after training as increase or decrease in connectivity. It is worth noting that the connectivity of the Sham group significantly differed from the Frontal tDCS (Occipital Cathodes) group in all three clusters, while it only differed from the Parietal tDCS group in two out of three, reflecting the behavioral outcome. Previous studies have shown that gains from WM training are correlated with altered connectivity in the brain, including clusters near the ones we found in the present study (Jolles et al., 2013; Kundu et al., 2013; Astle et al., 2015). Therefore, an interpretation is that the increased excitability of the neurons beneath the tDCS anodes interferes with the modification that would take place naturally, e.g., through LTP, during WM training, impairing the plasticity of the WM network. This effect could be exerted either through interfering with the strengthening of connectivity that would normally occur, perhaps via a cathode effect, or through strengthening of connectivity in networks other than the one expected, thereby altering the connectivity pattern.

The lack of a positive effect in our experiments should not be taken to imply that tACS or tDCS have a negative effect in general on cognitive performance or cognitive training. Rather, conclusions can only be drawn regarding the specific electrode positioning and stimulation parameters chosen in our experiment. Differences in setup between tES studies are a widespread problem, which complicates conclusions in the field. One difference in the present study compared to previous WM training studies with tES is the strength of the currents, which has been noted to give different results (Tremblay et al., 2014). However, in Experiment 1, we did see a significant impairment from 1 mA current, and it is unlikely that a higher current (as used in Martin et al., 2013; Richmond et al., 2014; Au et al., 2016) would have produced gains instead of impairments.

Moreover, although these WM training studies (Martin et al., 2013; Richmond et al., 2014; Au et al., 2016 and our study) included comparable samples of healthy young participants, they differed in their tasks for WM training. A variety of *n*-back, span and more complex tasks were used and stimuli were given in visual, auditory, or combined channels with spatial or verbal items to remember. These differences in behavioral paradigms could be part of the reason why different studies, including those presented in this manuscript, produce different results, e.g., both studies that reported gains during training used *n*-back tasks. Future studies could compare *n*-back and non-*n*-back WM training with identical stimulation set-up to test whether the specific task is responsible for the difference in effect of tES.

Furthermore, the negative effects found in this study highlight the concern of possible cognitive side effects of tES, a fact that has previously been discussed by Iuculano and Kadosh (2013). The spatial resolution of transcranial stimulation techniques is low, and the notion that tES can affect other cognitive networks apart from the intended one is quite self-evident (Iuculano and Kadosh, 2013). This limitation will hopefully be overcome with more widespread use of high-definition tDCS (Edwards et al., 2013).

A limitation of the current study is the small sample size and the short training regime (5 days), which might have limited our abilities to detect a positive effect of tES. However, similar group sizes and training lengths have resulted in positive effects of tDCS in training of numerical abilities (Cohen Kadosh et al., 2010). Furthermore, a significant effect of Day was found in all three experiments, which shows that the length of training was sufficient to measure reliable and significant improvements. Experiment 1 also showed that both group-size and training-time were sufficient to detect a significant impairment in training gains, and also affected resting state connectivity. All training groups showed lower gain than the sham group, although this was not always significant (**Figure 4**). In the light of these previous results and our evidences, we find it unlikely that larger group sizes or longer training time would have resulted in any positive effects.

## Conclusion

This study found that tDCS and tACS over the frontal or parietal lobes can have a negative effect on WM training gains. None of the stimulations applied had a positive effect. Moreover, the effect of tDCS on behavioral measures in Experiment 1 was paralleled by changes in resting state connectivity. Furthermore, the position of the cathodes was shown to affect the outcome of stimulation. Neuroimaging findings and negative or unexpected behavioral effects such as these are of particular importance in the light of recent interest in the general public around tES (Tremblay et al., 2014; Horvath et al., 2015), and as a contrast to an increasing amount of articles suggesting positive effects of tES.

In the light of the present results, it seems that bilateral stimulation of the frontal and parietal lobe is not more effective than unilateral stimulation, and can on the contrary impair training gain. This seems to be true both using tDCS and tACS. Overall, unilateral frontal stimulation regimes using higher current intensity than the one used in this paper (i.e., >1 mA) seem to be more effective than our stimulation paradigm and should be preferred in future interventions. Similarly, tACS intervention using theta frequency and stimulating frontal and parietal areas rather than bilateral frontal or parietal are to be preferred.

## Author Contributions

Study design: AM, FN, and TK; data collection: AM, FN, and KK; analysis: AM, FN, and TK; writing and editing: AM, FN, and TK.

## Conflict of Interest Statement

The authors declare that the research was conducted in the absence of any commercial or financial relationships that could be construed as a potential conflict of interest.

## Footnotes

1 http://nitrc.org/projects/artifact_detect/
